# A Focused Review of the Metabolic Side-Effects of Clozapine

**DOI:** 10.3389/fendo.2021.609240

**Published:** 2021-02-25

**Authors:** Jessica W. Y. Yuen, David D. Kim, Ric M. Procyshyn, William J. Panenka, William G. Honer, Alasdair M. Barr

**Affiliations:** ^1^ Department of Psychiatry, Faculty of Medicine, University of British Columbia, Vancouver, BC, Canada; ^2^ Department of Anesthesiology, Pharmacology and Therapeutics, Faculty of Medicine, University of British Columbia, Vancouver, BC, Canada

**Keywords:** antipsychotic, clozapine, cardiovascular disease, diabetes, metabolic syndrome, preclinical, side-effects

## Abstract

The second generation antipsychotic drug clozapine represents the most effective pharmacotherapy for treatment-resistant psychosis. It is also associated with low rates of extrapyramidal symptoms and hyperprolactinemia compared to other antipsychotic drugs. However, clozapine tends to be underutilized in clinical practice due to a number of disabling and serious side-effects. These are characterized by a constellation of metabolic side-effects which include dysregulation of glucose, insulin, plasma lipids and body fat. Many patients treated with clozapine go on to develop metabolic syndrome at a higher rate than the general population, which predisposes them for Type 2 diabetes mellitus and cardiovascular disease. Treatments for the metabolic side-effects of clozapine vary in their efficacy. There is also a lack of knowledge about the underlying physiology of how clozapine exerts its metabolic effects in humans. In the current review, we focus on key studies which describe how clozapine affects each of the main symptoms of the metabolic syndrome, and cover some of the treatment options. The clinical data are then discussed in the context of preclinical studies that have been conducted to identify the key biological substrates involved, in order to provide a better integrated overview. Suggestions are provided about key areas for future research to better understand how clozapine causes metabolic dysregulation.

## Introduction

Antipsychotic drugs represent the primary pharmacological treatment for schizophrenia spectrum disorders, and are increasingly used to treat other psychiatric conditions (1–4). Commonly categorized into first, second and third-generation drugs (5), the second-generation antipsychotics (SGAs) significantly improved quality of life by decreasing the incidence of neurological side-effects, such as extrapyramidal symptoms (EPS), that occurred with first-generation antipsychotic (FGA) drugs. However, SGAs are associated with higher rates of metabolic side-effects, which vary considerably by drug (6–10).

The SGA clozapine is the preferred drug for treatment resistant psychosis (11–15), producing therapeutic responses in approximately 30% of patients previously refractory to other antipsychotics (16–18), possibly reflecting a unique mechanism of action based on its complex pharmacology (19). It also has relatively low risk for EPS and hyperprolactinemia (20). Clinically, clozapine reduces violent and aggressive behavior in patients with schizophrenia (21, 22), and has been associated with the lowest all-cause mortality rate among all antipsychotics (23–25). Yet it is estimated that only 10–20% of eligible patients in the U.S. are prescribed clozapine, indicating that the drug is strongly underutilized (26).

This underutilization is due to a number of factors, including lack of prescribing experience by clinicians, institutional take-up of the drug, and concerns about blood monitoring for neutropenia/agranulocytosis, as well as other drug side-effects (27). With regards to the latter, clozapine is associated with a plethora of adverse effects (13, 26), which include a wide range of immune, metabolic, cardiovascular and psychiatric complications. Serious adverse effects include neutropenia/agranulocytosis (28), myocarditis/cardiomyopathy and tachycardia (29–31), and obsessive–compulsive symptoms (32, 33). However, the most common issues associated with clozapine use are the metabolic side-effects (34, 35), which occur in a majority of patients. These span a range of metabolic substrates, including glucose, insulin, lipids and body fat, which are collectively referred to as the “metabolic syndrome” (36). Patients who use clozapine consistently have more severe metabolic side-effects than with any other antipsychotic drug (6, 37). For example, a recent observational study of clozapine-treated outpatients noted that 80% were overweight, and 58% met criteria for metabolic syndrome, with concurrent high rates of hypertension, hyperglycemia and hyperlipidemia (38). Similarly, a retrospective chart review of clozapine users at community mental health clinics noted that 45% met criteria for metabolic syndrome, but these physical symptoms were often undertreated, with only 31% receiving treatment for hyperglycemia and 16% for hypertension (39). As premature death in schizophrenia is primarily caused by cardiometabolic disorders (40, 41), and clozapine often remains the only option for treatment resistant schizophrenia, it is imperative to understand in more detail the metabolic side-effects of clozapine use. In the present review, we summarize the main metabolic side-effects of clozapine in clinical populations, and integrate findings from recent preclinical studies to help elucidate the physiological pathways involved (42).

## Metabolic Syndrome

The clinical definition of metabolic syndrome has varied over past years depending on whether the emphasis was on insulin resistance, obesity or cardiovascular anomalies (36). In addition to Reaven’s initial description of hypertension, dysglycemia and dyslipidemia as factors that raise the risk for cardiovascular disease (CVD) and Type 2 diabetes mellitus (T2DM), the metabolic syndrome is also known to include abnormalities in coagulation and inflammation (43), and are frequently associated with obesity (44). Clinically, the metabolic risk factors themselves are not routinely measured for a diagnosis of metabolic syndrome. Instead, a diagnosis is made if three of the five following criteria are met: 1) waist circumference ≥ 102 cm in men and 88 cm in women (numbers change based on ethnicity), 2) triglyceride levels ≥ 150 mg/dl, 3) HDL cholesterol below 40 mg/dl in men and 50 mg/dl in women, 4) hypertension (blood pressure ≥ 130/85 mm Hg) and 5) glucose levels ≥ 100 mg/dl (45). Drugs used to treatment metabolic syndrome in clozapine users may target individual or multiple symptoms; for example, metformin is efficacious in concurrently ameliorating obesity, hyperglycemia and triglycerides (46).

## Abdominal Obesity

Obesity and weight gain, commonly estimated by the body mass index (47), potentially contribute to increased risk of cardiometabolic disorders. Previous studies have identified excess abdominal fat as an independent risk factor for CVD, where abdominal fat distribution in particular is a better predictor of CVD than body mass index (48). Of note, obesity does not necessarily coincide with insulin resistance, diabetes mellitus nor risk for CVD, since weight gain can be similar between patients, but visceral fat distribution can vary, thus emphasizing the importance of abdominal adiposity as an independent risk factor for cardiometabolic disorders in schizophrenia patients (49–51).

The propensity for clozapine to cause weight gain and obesity is well documented in patients with schizophrenia (52, 53). Compared to other antipsychotics, clozapine was associated with the largest amount of weight gain during the first 12 months of treatment and at up to 46 months, with 30.5% of patients subsequently developing T2DM (54). A follow-up study performed by the same authors revealed patients gained approximately 13.6 kg over a 10-year period of clozapine administration, with the risk of CVD and T2DM increasing over time in this cohort (41). Significant weight gain is a concern as an increase of >7% of desirable weight (the midpoint of a weight range for a specific height) can strongly predispose patients to risk for CVD (55, 56), which can only be partially reversed in clozapine treated patients by routine antidiabetic drugs, such as metformin (57). The specific risk factors associated with clozapine-induced weight gain, which varies considerably at the individual level, include variables such as sex, smoking status, and baseline levels of BMI, as well as interactions between these variables (58).

One of the key theories regarding the harms caused by fat accumulation, and visceral fat in particular, is the inflammatory hypothesis, in which clozapine-induced weight gain increases production of proinflammatory cytokines in insulin responsive cells, and monocyte infiltration and the inflammatory state contribute to insulin resistance (59). Cytokines and adipokines secreted by visceral white adipose tissue maintain metabolic and energy balance (60). Alterations in the levels of cytokines including interleukin 6 (IL-6) and tumor necrosis factor alpha (TNF-α), and adipokines such as adiponectin, leptin and resistin, have been associated with metabolic abnormalities (61, 62).

In particular, the extensively studied pro-inflammatory cytokine IL-6 is strongly correlated with all components of the metabolic syndrome in patients with schizophrenia (63). Increased plasma IL-6 levels are linked to obesity and reduced insulin sensitivity, *via* increased lipolysis, release of adiponectin and disruption of insulin signaling cascades (61, 64). TNF-α is also interconnected with obesity, T2DM and insulin resistance, where its expression is upregulated in obese individuals (65). TNF-α induces lipolysis through suppression of phosphodiesterase-3B and subsequently interfering with insulin’s antilipolytic effects (66). Alternatively, perilipin is subject to downregulation by TNF-α, resulting in increased access of hormone-sensitive lipase to triglycerides and increased lipolysis (67). Targeted mutations to abolish the function TNF-α receptors and expression of TNF-α improved insulin signaling in obese mice, suggesting TNF-α is critical for the development of insulin resistance (68, 69). Finally, inhibition of inositol receptor pathways by IL-6 and TNF-α leads to decreased glucose transport, thus resulting in insulin resistance (66).

Adipokines such as adiponectin, leptin and resistin originate from adipose tissue with important roles in metabolic homeostasis. Adiponectin has been extensively studied for its involvement in insulin sensitivity, obesity and T2DM (64). Circulating adiponectin levels are inversely related to risk for T2DM and metabolic syndrome and a promising therapeutic target (70). Adiponectin influences insulin sensitivity by activating 5’-AMP-activated protein kinase, leading to increased glucose uptake and free fatty acid oxidation (71). In obesity, the combined effects of reduced adiponectin and adiponectin receptor expression exacerbates insulin resistance and hyperinsulinemia (71, 72). Consequently, therapeutic strategies to manage metabolic syndrome and insulin resistance include raising adiponectin levels through increased release from adipocyte differentiation and gene transcription with the antidiabetic agent thiazolidinedione (TZD) (70, 73). Upregulation of adiponectin receptor expression is achievable by activation of peroxisome proliferator-activated receptor (PPAR)-α/γ (74, 75). Of note, PPAR-γ is also positively correlated with adiponectin levels in circulation, believed to be the result of directly affecting adiponectin production or its secretory mechanism (76). It was previously shown that the metabolic syndrome was more prevalent in patients treated with clozapine and this is associated with lower adiponectin levels (77).

Resistin is recognized as a connecting factor between insulin resistance, diabetes and obesity, albeit with debatable connections to the individual components of the metabolic syndrome (78, 79). Resistin is abundantly expressed in mononuclear cells and promotes inflammation, and raises the levels of cytokines such as TNF-α (78, 80). In contrast to its established pro-inflammatory effects, the evidence for resistin’s associations with obesity, insulin resistance and glucose regulation remains weak (78). Interest for resistin as a link between obesity and diabetes originated from the observations of increased insulin sensitivity in response to reducing resistin levels in obese mice (81). In humans, however, studies investigating resistin’s association with insulin resistance and T2DM are inconclusive and resistin has stronger implications in atherosclerosis and cardiovascular disease (80, 82, 83). While a causal role of resistin in metabolic anomalies remains to be determined, there is evidence of positive correlations between resistin and T2DM pathogenesis, which can largely be attributed to resistin’s pro-inflammatory properties (78, 84). Future studies focusing on neutralizing resistin in humans are warranted, especially when its native receptor remains to be identified in humans (84).

In addition, waist circumference is positively correlated with insulin resistance in nondiabetic patients with schizophrenia who received clozapine treatment (49). Of interest, increased waist circumference is the strongest predictor of insulin resistance among commonly used anthropometric measurements (e.g. body mass index, insulin sensitivity index, lipid levels) in clozapine-treated patients, but the same association was absent in patients treated with olanzapine (49).

Clozapine’s complex pharmacology and blockade of multiple receptors may contribute to this weight gain, where the histamine H_1_-receptor is believed to play a significant role (85). Weight gain occurs in H_1_-receptor “knock out” mice, in which high fat diets cause quicker body fat deposition compared to wild-type mice (86). Leptin potentially mediates the increase in adiposity, acting to disinhibit the negative feedback loop involving histamine neurons that normally suppress food intake (86) and increasing feeding. In support of this, animal experiments have shown clozapine reverses leptin’s effects *via* selective augmentation of hypothalamic adenosine monophosphate-activated protein kinase (AMPK) activity (87). As AMPK stimulation in the hypothalamus is known to increase food intake (88), clozapine potentially removes leptin’s anorexigenic effects by activating AMPK. In addition to suppressing appetite, decreased lipolysis resulting from inhibition of H_1_-receptors (89) may also contribute to clozapine-induced weight gain.

Clozapine also affects other hormones associated with obesity such as ghrelin and neuropeptide Y (NPY). Ghrelin serves to increase food intake by stimulation of ghrelin receptors located in brain centers involved in energy homeostasis, such as the hypothalamus and hindbrain (90). Chronically, ghrelin’s effects on adipogenesis, energy expenditure, lipolysis and dietary preference leads to the imbalance of energy intake and expenditure, eventually causing weight gain and potentially metabolic syndrome (90). Ghrelin is known to decrease as insulin levels rise in both rodents and humans, is correlated positively with insulin sensitivity, and has shown promise as a therapeutic target for diabetes (91, 92). Clozapine-treated patients have higher fasting serum ghrelin levels than control subjects, attributed to aberrant ghrelin secretion that is mediated through receptors such as the H_1_, serotonin (5-HT_2A/2C_) and dopamine (D_2_) receptors (93).

NPY also stimulates food intake and exogenous administration has demonstrated significant weight gain in rodents (94). NPY is elevated in obesity and promotes energy storage, and decreases in response to administration of leptin or insulin (94). Furthermore, it has been demonstrated that knockdown of the Y2 receptor for NPY can reduce abdominal fat and alleviate most symptoms of the metabolic syndrome (95). In rats, clozapine treatment elevates NPY levels in the arcuate nucleus of the hypothalamus involved in meal initiation (96). It has been shown polymorphisms in the *NPY* gene is associated with clozapine-induced weight gain and contributes to the development of obesity (97).

Clozapine-induced weight gain is potentially mediated through melanocortin receptor-4 (MC4R). MC4R is a downstream target of leptin signaling and mice lacking MC4R developed obesity (98). In humans, single nucleotide polymorphisms in the MC4R gene is associated with increased risk for weight gain in patients treated with clozapine and of European descent (99). Carriers of MC4R mutations display metabolic anomalies including increased energy intake, obesity and hyperinsulinemia, and the severity of these symptoms are correlated with the amount of functional MC4R (100).

Glucagon-like peptide-1 (GLP-1) is of worthy mention as a therapeutic intervention for obesity and T2DM resulting from clozapine treatment. GLP-1 acts to decrease food intake and glucagon secretion and increase insulin secretion, rendering it an attractive target for the management of metabolic disturbances (101). Importantly, rodent experimental data revealed clozapine decreased GLP-1 levels to raise glucose output and glucagon release and administration of GLP-1 agonists neutralizes these effects (101). A setback for the direct administration of GLP-1 is its rapid degradation, hence GLP-1 receptor agonists (GLP-1RAs) are preferred for prolonged glycemic and weight control (102). Preclinical studies using GLP-1RAs such as exendin-4 and Boc5 reversed glucose intolerance induced by clozapine treatment (103, 104). Human studies detailing the beneficial effects of GLP-1ARs in clozapine-treated patients are limited—a recent systematic review identified three studies demonstrating favorable outcomes on body weight, BMI, fasting glucose, waist circumference and BMI (105). Exenatide and liraglutide were used in these studies, with two studies showing weight loss following GLP-1AR administration (57, 106), and the other showing insignificant weight loss compared to controls (107).

While the above discussion is primarily centered around white adipose tissue, there is evidence that brown adipose tissue can mediate antipsychotic-induced weight gain, obesity and insulin resistance (60). Clozapine inhibits the differentiation of brown adipocytes and lipogenic genes (108), actions that are known to be associated with insulin resistance and energy balance (109). The downregulation of brown adipose marker uncoupling protein-1 (UCP-1) is of interest, as UCP-1 promotes leptin activity and possibly contributes to clozapine-induced weight gain through interference of insulin signaling (108).

## Dyslipidemia

Excessive plasma triglycerides ≥ 1.7 mmol/L and/or HDL-cholesterol levels below 40 mg/dl in men (<50 mg/dl in women), are part of the diagnostic criteria for the metabolic syndrome (36, 45). Dyslipidemia has detrimental effects on endothelial function and significantly increases the risk of coronary artery disease, particularly in individuals with diabetes (110). Endothelial injury arises through the accumulation of excessive lipids which eventually leads to atherosclerosis. Specifically, the infiltration of monocytes and T helper type-1 cells between dysfunctional endothelial cells results in the proliferation of smooth muscle cells and lipid-filled macrophages to form fibrous plaques characteristic of atherosclerosis (111).

Numerous studies have reported clozapine significantly raises serum triglyceride levels in patients (34, 35, 41, 112, 113). Treatment with clozapine over a 1-year period was associated with notable increases in serum triglycerides, and was significantly correlated with weight gain (114). The increase in serum triglycerides and total cholesterol occurred as early as the first month of initiating clozapine treatment and persisted throughout the study (114). However, other studies have noted that dyslipidemia can also occur independent of weight gain (113, 115). The causal association between clozapine treatment and hyperlipidemia is further confirmed in discontinuation studies, when increased triglyceride levels in clozapine-treated patients resolve follow switching to another antipsychotic (116). Of interest, elevated triglycerides in clozapine-treated patients are associated with improved outcome in patients with schizophrenia, as measured by decreased Positive and Negative Syndrome Scale (PANSS) scores (113, 117) and this is independent of weight gain (34). This raises the possibility of serum lipids influencing the pharmacokinetics and efficacy of clozapine (118), warranting further research, although it is unlikely clozapine causes changes in brain lipid levels (119, 120) which have been associated with clinical improvement.

Treatment options for clozapine-induced dyslipidemia include the use of statins that effectively lower cholesterol and triglyceride levels (121, 122). Considered the standard treatment for lowering cholesterol, statins lower LDL and total cholesterol levels, with a lesser effect on triglycerides (123). While displaying promising results for treating SGA-associated dyslipidemia, it should be noted statins can have adverse side effects. Notably, statins can elevate the risk of developing diabetes (124). Therefore, clinical benefits of improving cardiovascular health should be weighed against the increase in the incidence of T2DM from statin use, especially in patients treated with clozapine. The underlying mechanism for clozapine-induced dyslipidemia other than increased food intake remains unknown, and no confirmed receptor targets have been reliably identified. Given its involvement in cardiovascular function and regulating metabolism, the autonomic nervous system and its individual branches are potential candidates for mediating the cardiometabolic side effects of clozapine. In particular, heightened activity of the sympathetic nervous system contributes to glucose dysregulation and cardiovascular anomalies (125–127).

Alternatively, PPAR-α agonists are feasible candidates to manage clozapine-induced dyslipidemias. PPAR-α activation leads to improved HDL, LDL, triglyceride levels and has evident modulatory roles in energy homeostasis, as demonstrated in knockout mice that display hyperlipidemia and hypoglycemia (128, 129). Hypolipidemic fibrates are synthetic ligands for PPAR-α and are used to manage elevations in circulating triglycerides and decreased HDL cholesterol (129). In a case study, fenofibrate was administered to treat hyperlipidemia caused by clozapine treatment (130).

## Hypertension

Elevated blood pressure is common in both diabetic and obese individuals, present in 85% of patients diagnosed with metabolic syndrome (131). It was suggested the hypertension observed in these individuals is a result of compensatory mechanisms to the lack of response to insulin at the cellular level (132). Furthermore, poor response to insulin in insulin-resistant individuals is also a contributing factor, where insulin normally induces production of nitric oxide for vascular relaxation (133). Decreased insulin sensitivity leads to hyperinsulinemia as a compensatory mechanism, ultimately causing hypertension *via* activation of the renin angiotensin aldosterone system (131). Finally, an overactive sympathetic nervous system can lead to hypertension, often present in individuals with obesity and insulin resistance (134). Elevated plasma catecholamine levels induced by hyperinsulinemia may possibly contribute to the rise in blood pressure (135).

In comparison to the incidence rates of T2DM, dyslipidemia and obesity, there are fewer reported cases of hypertension in patients treated with clozapine (136). A claims-based approach study found no significant difference in the incidence of hypertension in patients treated with clozapine as compared to patients receiving FGAs (137). However, another chart review comparing patients treated with FGAs, SGAs (other than clozapine) or clozapine had contradictory results, where hypertension was strongly associated with clozapine use (136). At the end of the 5-year follow-up period, the clozapine group had significantly elevated blood pressure, resulting in 22% of these patients requiring medication for hypertension, as compared to 4% of the FGA group and 9% of the SGA group (136). Blood pressure increased as early as six months after initiating clozapine treatment, signifying the need to routinely monitor blood pressure as a prevention for CVD (136).

## Hyperglycemia

Hyperglycemia is the defining characteristic of metabolic dysfunction linked to T2DM (138). Individuals with fasting blood glucose levels between 5.6–6.9 mmol/l or 2-hour plasma glucose values of 7.8–11.0 mmol/l in the oral glucose tolerance test (OGTT) are considered to have impaired fasting glucose and impaired glucose tolerance, respectively (139). These individuals are considered to have elevated risk for developing T2DM, commonly known as the pre-diabetic stage. For diagnosis of T2DM, the standard biomarker for glycemic control, hemoglobin A1C, is commonly used. The A1C assay measures the indirect effects of plasma glucose levels over a span of 2–3 months and a value of ≥ 6.5% is used to diagnose T2DM (140). The A1C assay, coupled with a fasting plasma glucose of ≥ 7.0 mmol/l or a 2 h plasma glucose of ≥ 11.1 mmol/l in the OGTT, form the diagnostic criteria for diabetes (140).

Hyperglycemia causes cardiovascular damage by activating pathways that lead to excessive oxidative stress (141). Four mechanisms have been proposed to underlie glycemia-related vascular damage: decreased production of the antioxidant glutathione *via* activation of the polyol pathway, excessive generation of advanced glycation end products, activation of protein kinase C and increased activation of the hexosamine pathway (142). The common link between these four mechanisms is the inhibition of the glycolytic enzyme, glyceraldehyde-3 phosphate dehydrogenase (GADPH), by excessive superoxide production. Inhibition of GADPH results in increased upstream glycolytic intermediates and glucose, subsequently activating the aforementioned damaging pathways (142, 143). Several regions are susceptible to hyperglycemia-induced vascular damage, including the peripheral nerve, renal glomerulus and retina, as well as arteries in the brain, heart and lower limbs (141). Exposure to reactive oxygen species can adversely affect vascular contractile function, result in cardiomyopathy and atherosclerosis, and cause renal dysfunction (144), and clozapine was shown to cause oxidative stress in the liver in rats (145).

Clozapine has the highest propensity of all of the SGAs to induce hyperglycemia, which can usually can be resolved upon discontinuation (146–148). Glucose intolerance associated with clozapine treatment contributes to the development of new onset T2DM and exacerbates pre-existing cases (146, 149), both of which can occur independently of weight gain (148, 150). Weight gain is not present in all patients receiving clozapine treatment, and is consequently considered a contributing factor rather than the sole mechanism underlying insulin resistance (149, 151, 152). As a causal relationship between clozapine use and the development of diabetes mellitus cannot simply be attributed to excessive adiposity (153), there has been increasing attention given to weight-independent mechanisms to explain glucose dysregulation. One area of focus is clozapine’s antagonistic properties at receptors mediating glucose homeostasis, namely muscarinic, serotonergic and dopaminergic receptors (154). Acute antagonism of M_3_ and 5-HT_2A_ receptors, known to directly affect pancreatic β-cell function and insulin secretion (155), was found to decrease insulin secretion during the hyperglycemic clamp (which estimates peripheral insulin sensitivity and secretory capacity of β-cells following a glucose challenge) in animals whereas blockade of D_2_/D_3_ receptors had the opposite effect (154). In a follow up study, α_1_ antagonism with prazosin inhibited insulin secretion and glucose infusion rates after a glucose challenge, indicative of impaired β-cell function (156). As clozapine is known to rapidly reduce insulin sensitivity and alter hepatic glucose production (157), the role of antagonism of the above receptors as responsible for clozapine-induced impairment of β-cell function remains to be determined.

## Clozapine and Elevation of Plasma Catecholamines

The adrenoceptors and their endogenous ligands norepinephrine and epinephrine play a critical role in glucose homeostasis (127, 156, 158). Sympathetic activation rapidly raises blood glucose levels by suppressing insulin release, promoting glucagon secretion and hepatic gluconeogenesis and glycogenolysis *via* binding to G protein-coupled receptors (158). It is now well-established that clozapine treatment in both humans and animals causes a large increase in plasma levels of the these catecholamines. We recently reported the effects of multiple doses of the four different antipsychotic drugs haloperidol, risperidone, olanzapine, and clozapine on peripheral levels of the catecholamines dopamine, norepinephrine, and epinephrine in adult rats (159). While all drugs increased catecholamine levels, this effect was significantly larger in clozapine treated animals, and occurred with doses of the drug that we had previously shown to exert acute hyperglycemic effects (160–162).

Clinically, an earlier study noted that treatment with clozapine at 175–600 mg/day for 30 days resulted in a significant elevation of plasma norepinephrine levels, as well as heart rate, in patients with psychosis compared to healthy controls (163). Subsequent studies in patients with schizophrenia produced similar results (164–167). It was initially thought the increase in plasma norepinephrine was due to inhibition of α-adrenoceptors and the norepinephrine transporter, as the levels of the intraneuronal metabolite 3,4-dihydroxyphenylglycol (DHPG) remained unchanged (165). A follow up study refuted this hypothesis, because radiolabeled DHPG concentrations remained unchanged in plasma, and thus indicated normal reuptake and metabolism of norepinephrine (166). The authors suggested that increased norepinephrine vesicular fusion with the sympathetic nerve membrane accounts for the unchanged plasma DHPG levels and increased plasma norepinephrine. The mechanism through which clozapine elevates plasma norepinephrine, and whether this is associated with improved psychotic symptoms, remains moot (167). A possible reason for the discrepancy is the small sample numbers (n < 14) and short duration of available trials measuring plasma norepinephrine, with the longest published trial lasting 6 weeks (165, 167). However, the large increases in norepinephrine observed in these studies have potentially important implications not only for the metabolic side-effects of the drug, but also for the cardiovascular side-effects too, which we have described in detail previously (29).

## Preclinical Studies

In patients treated with antipsychotics, the causes of metabolic dysregulation are multifactorial, and include poor diet, lack of exercise, unhealthy habits (such as smoking/drinking) and direct effects of the antipsychotic itself (168). Teasing apart these individual contributions is challenging, and thus animal models of antipsychotic-induced metabolic dysregulation have provided key mechanistic insights (42), where the drug-specific effects can be studied separately. Preclinical studies with rodents have strong predictive validity, as the antipsychotics with the greatest metabolic liability in humans show similar effects in animals (162, 169–171). We would estimate that the most commonly studied antipsychotic is olanzapine (172–182), due to its potent metabolic effects and widespread use in patients. However, a number of preclinical studies have focused specifically on clozapine. These studies have demonstrated conclusively that treatment with clozapine can cause glucose intolerance, measured using the glucose tolerance test, and these effects are dose-dependent (104, 160–162, 183, 184). Importantly, these studies demonstrate that hyperglycemia occurs acutely, and is independent of weight gain (185). In a similar manner, a number of reports have examined the acute metabolic effects of clozapine using the hyperinsulinemic-euglycemic clamp, which is the “gold-standard” technique for measuring whole-body insulin resistance. Converging findings from different groups reliably show that clozapine causes profound insulin resistance (157, 186), and furthermore, the primary metabolite of the drug—norclozapine—also induces whole-body insulin resistance (160). The clamp studies implicate a direct effect of clozapine on increased hepatic glucose production and impaired beta cell function in the pancreas. The physiological mechanisms underlying these metabolic effects remain an ongoing area of study, but it has been suggested that clozapine’s effects on the autonomic nervous system may play a key role (187).

## Summary

It is now well established that treatment with clozapine is commonly associated with pronounced metabolic side-effects in many patients, typically greater than for all other antipsychotic drugs. These metabolic changes span a range of diverse metabolic substrates, leading to the development of metabolic syndrome and ultimately T2DM and CVD in many patients. These sequelae contribute to underutilization of the drug, which represents a serious concern, as clozapine is uniquely efficacious in managing treatment resistant psychosis. Treatment of these metabolic changes is only partly effective in most cases, and so a better understanding of the physiology may be required to develop more effective interventions. Animal models of clozapine’s metabolic side-effects have already provided important insights into how the drug directly affects metabolic physiology, and may be used to help identify novel pharmacotherapies when working in parallel with clinical studies.

## Author Contributions

All authors contributed to the article and approved the submitted version.

## Funding

This work was supported by a Natural Sciences and Engineering Research Council of Canada grant to AB, and a British Columbia Provincial Health Services Authority grant to AB and RP. WH was supported by the Jack Bell Chair in Schizophrenia.

## Conflict of Interest

WP reports personal fees from Abbatis Bioceuticals, Medipure Pharmaceuticals and is owner of Translational Life Sciences. RP reports personal fees from Janssen, Lundbeck and Otsuka. WH reports personal fees from Canadian Agency for Drugs and Technology in Health, AlphaSights, Guidepoint, Translational Life Sciences, Otsuka, Lundbeck, and Newron, grants from Canadian Institutes of Health Research, BC Mental Health and Addictions Services, and has been a consultant (non-paid) for In Silico. AB has been a scientific advisor to Emerald Health Therapeutics, Cannevert Therapeutics, Global Cannabis Applications Corp, Medipure Pharmaceuticals, Vitality Biopharma and Oakum Cannabis Corp.

The remaining authors declare that the research was conducted in the absence of any commercial or financial relationships that could be construed as a potential conflict of interest.
